# Adenosine and Immune Imbalance in Visceral Leishmaniasis: The Possible Role of Ectonucleotidases

**DOI:** 10.1155/2012/650874

**Published:** 2011-10-05

**Authors:** Rafael Paletta-Silva, José Roberto Meyer-Fernandes

**Affiliations:** ^1^Instituto de Bioquímica Médica, Universidade Federal do Rio de Janeiro, CCS, Bloco H, Cidade Universitária, Ilha do Fundão, 21941-590 Rio de Janeiro, RJ, Brazil; ^2^Instituto Nacional de Ciência e Tecnologia de Biologia Estrutural e Bioimagem (INCTBEB), CCS, Bloco H, Cidade Universitária, Ilha do Fundão, 21941-590 Rio de Janeiro, RJ, Brazil; ^3^Laboratório de Bioquímica Celular, Instituto de Bioquímica Médica, Centro de Ciências da Saúde, Universidade Federal do Rio de Janeiro (UFRJ), Cidade Universitária, Ilha do Fundão, 21941-590 Rio de Janeiro, RJ, Brazil

## Abstract

Visceral leishmaniasis (VL) is the most severe form of leishmaniasis and is responsible for most *Leishmania*-associated deaths. VL represents a serious public health problem that affects many countries. The immune response in leishmaniasis is very complex and is poorly understood. The Th1 versus Th2 paradigm does not appear to be so clear in visceral leishmaniasis, suggesting that other immunosuppressive or immune-evasion mechanisms contribute to the pathogenesis of VL. It has been demonstrated that generation of adenosine, a potent endogenous immunosuppressant, by extracellular enzymes capable to hydrolyze adenosine tri-nucleotide (ATP) at the site of infection, can lead to immune impairment and contribute to leishmaniasis progression. In this regard, this paper discusses the unique features in VL immunopathogenesis, including a possible role for ectonucleotidases in leishmaniasis.

## 1. Introduction

Parasites that belong to the genus *Leishmania* are among the most diverse human pathogens, both in terms of geographical distribution and in the variety of infection-induced clinical manifestations they generate. Of the 88 countries affected by leishmaniasis, 72 are classified as developing countries, including 13 of the least developed countries. Despite the widespread distribution of leishmaniasis, 90% of VL cases occur in just five countries: Bangladesh, India, Nepal, Sudan, and Brazil [1]. *Leishmania* are obligate intracellular protozoan parasites transmitted by phlebotomine sand flies. Flagellated promastigotes injected by sand fly bites replicate as intracellular amastigote stages inside mononuclear phagocytic cells of mammalian hosts [2, 3]. The epidemiology of leishmaniasis is diverse, with 20 *Leishmania *species that are pathogenic in humans and 30 sand fly species that have been identified as vectors [1]. This range of species variability culminates in different symptomatology and clinical manifestations that can be classified into two broad disease presentations: cutaneous leishmaniasis and VL [4]. 

Visceral leishmaniasis (VL) is caused by *Leishmania donovani *(*L. donovani*) in the Indian subcontinent, Asia, and Africa and by *Leishmania infantum *(*L. infantum*) or *Leishmania chagasi *(*L. chagasi*) in the Mediterranean region, southwest and central Asia, and South America; however, other species, such as *Leishmania amazonensis* (*L. amazonensis*) in South America, are occasionally viscerotropic [5, 6]. VL is the most severe form of leishmaniasis and is responsible for most deaths caused by leishmaniasis. The disease commonly affects the liver, spleen, and lymph nodes. The pathophysiology of the disease is characterized by the onset of symptoms of a persistent systemic infection, including loss of appetite, weight loss, intermittent fever, fatigue, and exhaustion. The incubation period usually ranges from two to six months. Parasite dissemination occurs via the vascular, lymphatic, and mononuclear phagocyte systems and results in bone marrow infiltration, hepatosplenomegaly, and lymphadenopathy [7, 8].

In VL, the immune response is closely related to disease progression and the development of clinical manifestations. The syndromes and causative mediators are typical of a slowly developing systemic inflammatory response syndrome with multiorgan failure. The absence of parasite actions or products that would harm the host cells or tissues is a further indication that the systemic pathogenicity of VL is dependent on the host response [9].

## 2. Immune Response in Visceral Leishmaniasis: The Th1 versus Th2 Paradigm

Cytokine production and the subsequent stimulation or inactivation of certain immune cells in the early stages of an infection essentially determines the type of immune response [10]. The complexity of the immunological events triggered during active VL and the relevance of the segregation of human immune responses during VL into type 1 and type 2 still remain unclear. Several studies have identified that the major immunologic dysfunction observed in VL is the inability of T cells to produce IL-2 and IFN-*γ* upon stimulation with *Leishmania *antigens and to activate macrophages and kill *Leishmania* parasites. Analysis of Th1 and Th2 cell-associated cytokines in peripheral blood samples from patients with *L. donovani*-induced VL showed reduced IFN-*γ* levels and increased IL-4 levels in comparison to healthy patients in the control group [11].

Recent data have challenged the simplicity of Th1 versus Th2 model and revealed further complexities in cytokine regulation and the mechanisms of acquired resistance and immune escape [3]. For some cases of VL, it appears that the immune response is a mixed Th1 and Th2 type. In patients with VL, although the production of type 1 cytokines was not depressed, cells appeared to be unresponsive to stimulation with type 1 cytokines [10]. Increased production of multiple cytokines and chemokines in VL patients was also observed; the response was predominately proinflammatory, as indicated by the elevated plasma levels of IL-1, IL-6, IL-8, IL-12, IL-15, IFN-*γ*-inducible protein-10 (IP-10), monokine induced by IFN-*γ* (MIG), IFN-*γ*, and tumor necrosis factor (TNF)-*α* [2, 12]. Gene expression analysis in an *in vitro* model, using human monocyte-derived macrophages (MDMs) challenged with *L. chagasi* promastigotes that were subsequently cocultured with or without *Leishmania*-naïve autologous T-cells, found that the initial encounter between *L. chagasi* and cells of the innate and adaptive immune system primarily stimulated type 1 immune cytokine responses. Type 1 cytokine responses were produced despite a lack of classical macrophage activation, suggesting that the local microenvironment at the site of parasite inoculation may determine the initial course of T-cell differentiation [13]. 

Investigations into the mechanisms underlying the immunosuppression observed during acute VL have demonstrated defective antigen-specific proliferation and IFN-*γ* responses [14], which suggest that the parasites suppress macrophage microbicidal responses, and IFN-*γ* signaling pathways [15] at the earliest stages of infection. Also supporting the absence of a clear Th1 versus Th2 dichotomy in VL is the observation that inhibition of Th1 cytokines in a murine model of *L. chagasi*-induced VL results in parasite clearance independent of Th2 cytokines [16]. Studies, not only in mice, but also in humans, suggested that cure was independent of the differential production of Th1 and Th2 cytokines, and both IFN-*γ* and IL-4 producing T cells have been isolated from asymptomatic and cured patients [17]. These immunosuppressive mechanisms could differ depending upon etiologic agent, T-cell subpopulation predominance at site of infection, stage of infection, and target organ. Such mechanisms could be even more complex because the course *Leishmania* infection may vary widely depending on the species and strain of parasite.

## 3. Immunomodulatory Effects of Adenosine and Adenosine Triphosphate (ATP) Nucleotidases

In studying the complexity of immune system regulation, especially at the site of injury, several groups have recently begun to demonstrate a possible role for the nucleoside adenosine and adenosine nucleotidases in regulating *Leishmania*-specific immune responses. It was proposed that ecto-ATP diphosphohydrolases and ectonucleotidases, which are membrane-associated enzymes with their catalytic sites turned extracellular, would act to dephosphorylate adenosine nucleotides into free adenosine [18, 19]. Before discussing the role of ectonucleotidases in leishmaniasis, concepts and comments regarding the immunomodulatory roles of adenosine nucleotides and nucleosides must be stressed.

Extracellular nucleotides are involved in a variety of physiological functions by participating in extracellular signaling through the activation of cell surface metabotropic purinergic (G protein coupled) and ionotropic (ion channel coupled) receptors. The major classes of purinergic receptors include the type P2 receptors (P2X-P2Y) and ionotropic and metabotropic receptors [20–22]. The activation of these receptors by ATP leads to proinflammatory effects by initiating a response characterized by the secretion of IFN-*γ*, IL-12, and TNF-*α* [23].

During acute inflammation, the ATP released by the leakage of cellular contents can be sequentially dephosphorylated by the action of ectonucleotidases, causing the concentration of extracellular adenosine to increase markedly [22, 24]. Adenosine exerts distinct effects on the immune system compared to ATP. The immunosuppressive actions of adenosine are triggered by activation of four receptor subtypes (A_1_, A_2A_, A_2B_, and A_3_) belonging to the family of purinergic receptors known as P1 or A. These receptor subtypes are members of a superfamily of receptors composed of seven transmembrane regions that are coupled to G proteins [22, 25] and expressed in neutrophils, macrophages, dendritic cells [26], and T cells [27]. These four adenosine receptor subtypes are expressed concomitantly in various immune cells [28, 29].

Purinergic signaling depends on several factors, including receptor expression, receptor sensitivity, and the levels of extracellular nucleotides and nucleosides. The concentration of extracellular adenosine can determine the activation of a given receptor due to the difference in A receptors affinity [28]. In physiological adenosine concentrations (0.2–0.5 *μ*M), the A_1_ and A_3_ receptors are preferentially activated, while at higher adenosine concentrations (16,2–64,1 *μ*M), such as those seen in inflammation, A_2B_ receptors exert their effects [30]. 

**Figure 1:**
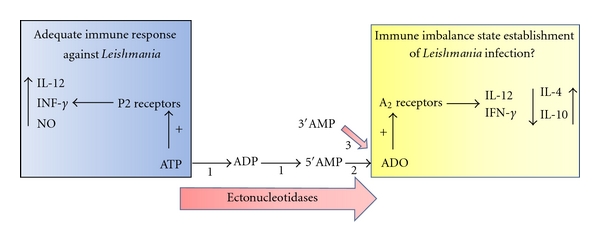
Partial reactions catalyzed by ecto-nucleoidases: (1) ecto-ATPase, ectonucleoside triphosphate diphosphohydrolase; (2) ecto-5′-nucleotidase; (3) ecto-3′-nucleotidase. The sequential hydrolysis of extracellular ATP by *Leishmania* ectonucleotidases triggers the host inflammatory and immune response following P2 receptor activation by extracellular ATP (blue square) and P1 (A_2_) receptors activation by extracellular adenosine nucleoside (yellow square). ADO: Adenosine.

Activation of A_2A_ and A_2B_ receptors leads to increased levels of intracellular cAMP by activating adenylyl cyclase, which inhibits immune cell function [31]. This regulatory effect is caused mainly by inhibiting the production of IFN-*γ*, IL-12, and TNF-*α* coupled with an increased production of IL-10 [32] as summarized in Figure 1. Inhibition of monocyte maturation and the suppression of macrophage phagocytic function are also related to the activation of A_2_ receptors [33]. The signaling pathway that couples A_2A_ and A_2B_ adenosine receptors with G proteins is balanced by the signaling that results from the association of A_1_ and A_3_ receptors with Gi protein, which inhibits adenylyl cyclase. Adenylyl cyclase inhibition then leads to decreased levels of cAMP, which limits the premature inhibition of immune cells by A_2_ receptors [34].

## 4. Adenosine and the Establishment of *Leishmania* Infection: The Possible Role of Ectonucleotidases in Immune Impairment

Ectonucleotidases are glycoprotein enzymes present in the plasma membrane with their catalytic sites facing extracellularly [18, 19], which are capable of hydrolyzing extracellular nucleotides. Fundamentally important in maintaining the homeostasis of extracellular nucleotides, these enzymes are also regulatory (termination of signaling events triggered by ATP) and metabolic (generation of nutrients) in function [35]. The hydrolysis of extracellular nucleotides occurs in a sequential manner by the action of ecto-ATPase, ecto-ADPase, ecto-ATPDase, and ecto-5′-nucleotidase [36]. The classification of ectonucleotidases in different families takes into account kinetic aspects such as enzyme specificity and substrate affinity and molecular aspects of protein structure [37]. 

Since Gottlieb and Dwyer [38] provided information in 1981 about the biochemistry of surface membranes of *Leishmania*, a large number of authors have observed nucleotidases located on the outer surface of the plasma membrane of the parasite. For example, ecto-ATPases, ecto-5′-nucleotidase, and ecto-3′-nucleotidase have all been described in *Leishmania *sp. [18, 19, 39, 40]. 

In *Leishmania *parasites, it was proposed that an increase in ectonucleotidase activity would increase the production of adenosine, which would consequently aid in the establishment of infection through its immunosuppressive mechanisms. Infective *L. amazonensis* promastigotes exhibit higher ATPase activity compared to nonvirulent promastigotes [39]. Furthermore, amastigotes, which are responsible for maintenance of leishmaniasis in the vertebrate host, are capable of hydrolyzing ATP at higher rates than promastigotes [40]. *L. amazonensis* strains that possess higher ectonucleotidase activity are more effective in establishing infection in murine models [41]. Comparison of ectonucleotidase activities between *L. amazonensis*, *Leishmania braziliensis* (*L. braziliensis*), and *L. major* showed that the more virulent parasite causing nonhealing lesions in C57BL/6 mice (e.g., *L. amazonensis*) hydrolyzes higher amounts of ATP, ADP, and 5′AMP [42]. Furthermore, adenosine treatment at the time of *L. braziliensis* inoculation delays lesion resolution and induces increased parasite burdens. Consistent with this, inhibition of adenosine receptor A_2B_ led to decreased lesion size and lower parasite burden [43]. In clinical practice, results that are consistent with the idea that ecto-ATPase activity is involved in virulence were also observed, whereby *L. amazonensis* strain isolated from a human case of VL possess higher ecto-ATPase activity than strains isolated from CL cases [44].

In addition to these ectonucleotidases, *Leishmania* parasites also express a bifunctional enzyme called 3′-nucleotidase/nuclease (3′NT/NU) in the plasma membrane with a high capacity to hydrolyze 3′ribonucleotides and nucleic acids [43, 45]. Although first identified in *L. donovani* [43, 45], it was later found in *L. chagasi* [46], *L. major* [47], *L. mexicana *[48], and *L. amazonensis*. Although the 3′-nucleotidase enzyme is found solely in some trypanosomatids, 3′-nucleotides are available through nucleic acid hydrolysis in several mammalian tissues [49], especially the spleen, an organ commonly targeted by *Leishmania* parasites. Recently, our group has characterized the 3′-nucleotidase activity of *L. chagasi* and demonstrated that such activity could be related to aspects of parasite virulence [46]. Interestingly, the viscerotropic *L. chagasi* and *L. donovani* had higher 3′-nucleotidase activity compared to the New World and Old World dermatotropic species (e.g.,* L. amazonensis*, *L. major*, *L. tropica,* and *L. braziliensis*). *L. chagasi* metacyclics (infective promastigote stage) had higher 3′-nucleotidase activity compared to *L. chagasi* nonmetacyclics (noninfective promastigote stage) [46]. Similar results were also observed with *L. amazonensis*, where infective promastigotes possessed twofold higher 3′-nucleotidase activity compared to nonvirulent promastigotes [50]. 

In addition to the role of ectonucleotidase in the establishment of *Leishmania* infection by ectonucleotidases, some interesting data have further demonstrated that high expression/activity of such enzymes on immune cells contributes to immune imbalance. CD4^+^CD25^+^ regulatory T cells (Tregs) from mutant mice deficient in CD39 have impaired regulatory function manifesting as a 50% decrease in the ability of CD39-null Tregs to modulate effector T-cell function *in vitro* and *in vivo*. These results indicate that CD39 expressed by Treg is the major and rate-limiting ectonucleotidase responsible for the generation of adenosine and suggest that a putative CD39/CD73-adenosinergic axis may contribute to the immunoregulatory function of Treg [51, 52]. 

In *L. infantum*-infected BALB/c mice, Tregs are present. The high levels of Foxp3 gene expression and surface expression of Eb7 integrin (CD103) suggest a predisposition for Treg retention within sites of *L. infantum* infection, as is the case of the spleen and draining lymph nodes, consequently influencing local immune response [53]. It was attributing to CD4^+^CD25^+^FOXP3^+^ regulatory T cells an important role in immune suppression because in those cells, CD39 and CD73 are overexpressed. Inhibitors of ectonucleotidase activities and antagonists of the A_2A_ receptor blocked Treg-mediated immunosuppression [54]. Consistent with this view, it was demonstrated that patients with VL possess high levels of adenosine, which was related to ectonucleotidase activities and disease progression [55]. 

This paper about the immune modulatory effects of adenosine and the production of such nucleoside by parasite and immune cells provide new perspectives for the understanding of the complex immune response in leishmaniasis. Further studies are needed, especially using visceral leishmaniasis models, to clearly delineate the role of ectonucleotidases in the establishment of infection and *Leishmania*-induced immunomodulation.
